# Statistical evaluation of growth parameters in biofuel waste as a culture medium for improved production of single cell protein and amino acids by *Yarrowia lipolytica*

**DOI:** 10.1186/s13568-020-00968-x

**Published:** 2020-02-18

**Authors:** Monika Elżbieta Jach, Tomasz Baj, Marek Juda, Robert Świder, Barbara Mickowska, Anna Malm

**Affiliations:** 1 Department of Molecular Biology, The John Paul II Catholic University of Lublin, 1i Konstantynów Street, 20-708 Lublin, Poland; 2 Chair and Department of Pharmacognosy, Medical University of Lublin, 1 Chodzki Street, 20-093 Lublin, Poland; 3 Department of Pharmaceutical Microbiology, Medical University of Lublin, 1 Chodzki Street, 20-093 Lublin, Poland; 4 Faculty of Food Technology, Malopolska Centre of Food Monitoring, The Agricultural University of Cracow, Balicka Street 122, 30-149 Cracow, Poland

**Keywords:** Amino acids, Biofuel waste, Fermentation optimization, Single cell protein, *Yarrowia lipolytica*

## Abstract

*Yarrowia lipolytica* is an oleaginous yeast species with the ability to grow on a number of substrates types, especially industrial wastes. This paper concerns the statistical optimization of fermentation parameters and media to ensure consistent and improved *Y. lipolytica* protein production. A strain of *Y. lipolytica* A-101 was observed to be proficient in producing single cell protein, amino acids, and vitamin B12 while utilizing biofuel waste instead of a complete YPD medium for yeast growth. A fractional fractal design experiment was then applied, and the two fermentation parameters of temperature and pH were recognized to have a significant effect on the protein and amino acid production. Subsequently, the response surface methodology with a three-level complete factorial design was employed to optimize these influential parameters. Therefore, five different measuring systems were utilized to construct a quadratic model and a second-order polynomial equation. Optimal levels of parameters were then obtained by analysis of the model and the numerical optimization method. When the *Y. lipolytica* A-101 was cultivated at optimized pH (5.0) using biofuel waste as a medium, the protein concentration was increased to 8.28—a 44% enhancement as compared to the original (3.65). This study has thus demonstrated a beneficial way to cultivate *Y. lipolytica* A-101 on biofuel waste for enhanced production of single cell protein and amino acids for use in human diet and in animal feed.

## Introduction

*Yarrowia lipolytica* is one of the better-known oleaginous yeasts with the ability to grow on a variety of hydrophilic and hydrophobic substrates. These can be conventional (starch, molasses, fruit, and vegetable wastes) and non-conventional (different fractions of petroleum, natural gas, ethanol, methanol, lignocellulosic biomass, waste cooking or motor oils, animal-waste fats, or waste streams from various industries). Because of such inexpenside feedstocks, microbial mass and microbial lipid produced by* Yarrowia lipolytica* (single cell protein and single cell oil) are considered as environmentally friendly processes (Dourou et al. 2018; Groenewald et al. 2013; Jach et al. 2017; Jach and Serefko 2018; Katre et al. 2012; Lopes et al. 2018, 2019; Papanikolaou et al. 2001, 2003; Rywinska et al. 2013).

*Yarrowia lipolytica* is a rich natural source of various nutritional components (peptides, trace minerals, fats, especially mono-unsaturated fatty acids (MUFAs) and saturated high added-value lipids like cocoa-butter equivalents). Of note, it can accumulate lipids intracellularly to ≥ 40% of its dry cell weight. Furthermore, the biomass of *Y. lipolytica* contains essential amino acids (e.g. lysine and methionine) that are present only in limited quantity in most plant and animal foods (Adedayo et al. 2011; Bellou et al. 2016; Beopoulos et al. 2011; Dourou et al. 2018; Jach et al. 2017; Lopes et al. 2018, 2019; Papanikolaou et al. 2001,2003; Rywinska et al. 2013; Zhao et al. 2016). This yeast also has the ability to accumulate vitamin B12 into its cells from biofuel waste used as a medium in a similar manner to animal cells (Jach et al. 2018).

Since this yeast contains a variety of nutrients in its biomass, it is deemed nutritional yeast. *Y. lipolytica* is safe and nonpathogenic to humans. It is found as living cells in many processed foods such as cheese, mayonnaise, and meat (Groenewald et al. 2013). Living cells of *Y. lipolytica* are a rare opportunistic fungal pathogen only in patients with compromised immunity and those with catheters (Zieniuk and Fabiszewska 2019). Indeed, several production processes utilizing *Y. lipolytica* have been granted the “Generally Regarded as Safe (GRAS)” status by the US FDA (Groenewald et al. 2013; Zieniuk and Fabiszewska 2019). Nutritional *Y. lipolytica* biomass can only be added to the regular human diet after killing cells by drying to help solve the problem of food shortages in rapidly growing populations, especially in developing countries like India (Jach et al. 2017; Jach and Serefko 2018).

Currently, *Y. lipolytica* is used as nutritional biomass for feeding livestock and as a biotechnological production host for organic acids, e.g. citric acid or hydrophobic substances like carotenoids or PUFAs. It is also employed as a heterologous production host for pharmaceutical and industrial proteins and enzymes as well as for bioremediation purposes (Groenewald et al. 2013). In 2010, *Y. lipolytica* biomass obtained from biofuel production waste was authorized by the European Feed Manufacturers’ Federation as a feed additive (Jach et al. 2018). It is worth to mention that yeast grows faster than plants or animals and produces large quantities of important nutrients, e.g. it accumulates vitamin B12 in its biomass, from a relatively small area of land and within a short time (Jach et al. 2017; Jach and Serefko 2018; Jach et al. 2018). Thus, *Y. lipolytica* occupies a considerable place in pharmaceutical, environmental protection, feed and food industries (Jach and Serefko 2018; Lopes et al. 2019).

The use of inexpensive waste components is critical for production of cheap nutritional yeast biomass. In the field of yeast biomass, much effort has been directed toward optimizing production. With regard to this, production of yeast biomass can be improved by optimizing the physical parameters and nutritional constituents of the employed medium.

Optimization experiments are performed using statistical experimental design approaches. Such experimental design techniques are used to select significant variables in order to obtain optimal levels. The application of these statistical experimental design techniques in medium optimization can result in improved yields, reduced process variability, and reduced time and overall cost, in comparison to conventional practice of single factor optimization (Polpass et al. 2013; Shahabadi and Reyhani 2014).

Our project assessed the potential of biofuel waste for the production of *Yarrowia lipolytica* A-101 as a source of nutritional biomass for human intake. In doing this, we showed (using statistical techniques) that *Y. lipolytica* grown in biofuel waste produced biomass rich in SPC and amino acids (Jach et al. 2017). We then implemented the results from statistical research into culture optimization of biofuel waste in order to develop a successful optimization strategy and to discern the physical parameters that significantly influence protein-enriched biomass production.

## Materials and methods

### Microbial strains

We used wild type yeast *Yarrowia lipolytica* A-101 obtained from Skotan S.A. Poland, whereas the reference yeast strain *Yarrowia lipolytica* ATCC 9793 was obtained from LGC Standards.

### Production, *Y. lipolytica* biomass harvesting, and variations of fermentation parameters

*Yarrowia lipolytica* was cultured on two trial media: standard YPD medium (Difco) and industrial SK medium, as previously described (Jach et al. 2017). This latter is waste from biofuel (biodiesel) production (a mixture of vegetable oils, degumming and glycerol fractions formed during biofuel production). Biofuel is made through chemical reaction of vegetable oil with ethanol producing fatty acid esters (long-chain alkyl (methyl, ethyl, or propyl) esters). The SK medium was provided by Skotan S.A. (Poland). In our work, the standard biofuel production waste was replaced with a partially refined, desalinated, and methanol-free by-product from a biodiesel manufacturer (from Lotos Group refineries, Poland, to Skotan S.A.). Before sterilization in all media in Erlenmeyer flasks (150 ml), pH was adjusted to 5.0, 6.0, and 7.0 using 1 N HCL and/or 1 N NaOH, respectively. On the laboratory scale, fermentation was carried out at 20, 25, or 30 °C at 200 rpm in an incubator shaker flask by determination of biomass and other parameters after 12- or 18-h intervals.

### Determination of the concentration of SCP and amino acids in yeast biomass

Chemical analyses were carried out as previously described (Jach et al. 2017).

### Statistical analysis of data

The fractional factorial design (FFD) was employed for screening the most significant physical parameters for growth and protein-enriched biomass production. FFD was used to obtain a combination of values that can optimize the response within the region of the two dimensional observation spaces. This facilitates designing a minimal number of experimental runs (Atkinson 2014; Berger et al. 2018; Esfe et al. 2015, 2017; Polpass et al. 2013; Shahabadi and Reyhani 2014). Statistica, v. 12 Software (StatSoft, Inc, Tulsa, OK, USA; 2012) was used for the evaluation of experimental data.

In the experimental design, two independent parameters (explanatory variables) were screened: temperature (Temp.,  °C) in a range from 20 to 30 °C and pH values from 5.0 to 7.0. The dependent variables (response) included total nitrogen (TN) and total protein (TP) for both strains. Table 1 shows the experimental parameters and corresponding codes (− 1, 0, and + 1).

**Table 1:** Optimalization of physical parameters for the protein yeast biomass production: independent variables in a 2^2^ full factorial experiment design

Independent factor	Actual factor level at coded factor level of			
	Symbol	− 1	0	1
Temperature (°C)	X_1_	20.0	25	30
pH	X_2_	5.0	6.0	7.0

The parameters used in this experiment are temperature and pH

The statistical significance of the model was verified by applying the analysis of variance (ANOVA). All data is expressed as a mean ± SD (standard deviation) of three independent experiments. The differences between the concentrations of SCP in biomass obtained in the different conditions were compared to *Y. lipolytica* A-101 cultivated on the YPD medium at 30 °C and pH 6.0, using two-sided student’s *t*-test in Statistica software version 12.0 (StatSoft, Inc, Tulsa, OK, USA; 2012). The *P* value < 0.05 was considered statistically significant.

Tables 2 and 3 show the details of the design and the experimental and approximated results obtained using the system of a multivariable regression model. The behavior of the system was explained by Eq. (1) (Matos et al. 2015) with a slight modification that includes linear and quadratic effects:

1 $$Y = {\beta _0} + {\beta _1}{{\text{X}}_1} + {\beta _2}{{\text{X}}_2} + {\beta _{11}}{{\text{X}}_1}^2 + {\beta _{22}}{{\text{X}}_2}^2$$

where *Y* is the predicted response, *β*_0_ is the intercept term, *β*_1_, *β*_2_ are the linear coefficients, *β*_11_, *β*_22_ are the quadratic and the interaction coefficient, and X_1_, X_2_ represent the independent variables (temperature and pH, respectively). The model evaluates the effect of each independent variable to the response. The analysis of the factorial planning consists of quantifying the effect of the factors on one determined response. The studied variables were the temperature and pH (minimum and maximum) (Table 1) corresponding to the TN and TP variables, respectively, in the assessment of protein production by both *Y. lipolytica* strains.

**Table 2:** The average values of the nitrogen and protein in wet biomass obtained in cultivation of *Yarrowia lipolytica* strains on biofuel waste or YPD medium in relation to temperature and pH

No	Independent variables		Biofuel waste								YPD medium							
			TN (ATCC)		TN (A101)		TP (ATCC)		TP (A101)		TN (ATCC)		TN (A101)		TP (ATCC)		TP (A101)	
	Temp. (°C)	pH	Mean	SD	Mean	SD	Mean	SD	Mean	SD	Mean	SD	Mean	SD	Mean	SD	Mean	SD
1	20.0	6.0	0.80	0.04	0.67	0.01	5.01	0.27	4.19	0.08	1.45	0.09	1.53	0.03	8.97	0.45	9.60	0.16
2	25.0	6.0	0.72	0.26	0.53	0.02	4.53	1.63	3.28	0.10	1.37	0.05	1.67	0.11	8.55	0.29	10.40	0.68
3	30.0	5.0	0.00	0.00	*1.32*	0.18	0.00	0.00	*8.28*	1.11	1.87	0.21	*1.85*	0.09	11.66	1.34	*11.57*	0.55
4	30.0	6.0	*1.14*	0.17	0.58	0.20	*7.09*	1.05	3.65	1.25	*1.93*	0.21	1.57	0.07	*12.03*	1.28	8.39	1.58
5	30.0	7.0	0.77	0.30	0.77	0.29	4.15	1.95	4.84	1.81	1.50	0.14	1.54	0.15	9.35	0.87	9.60	0.95

TN, total nitrogen; TP, total protein; ATCC, *Y. lipolytica* ATCC 9793; A-101, *Y. lipolytica* A-101

**Table 3:** Experimental design with experimental and predicted values of protein concentration in wet biomass of *Yarrowia lipolytica* strains obtained in cultivation on biofuel waste (SK medium)

Code			Biofuel waste							
			TN (ATCC)		TP (ATCC)		TN (A-101)		TP (A-101)	
No.	X_1_	X_2_	Exp.	Pred.	Exp.	Pred.	Exp.	Pred.	Exp.	Pred.
2	− 1	0	0.77	0.80	4.8	5.01	0.66	0.67	4.13	4.19
5	0	0	0.92	0.72	5.77	4.53	0.51	0.53	3.16	3.28
7	1	− 1	0.00	0.00	0.00	− 0.00	1.12	1.32	7.02	8.28
8	1	0	1.21	1.14	5.89	7.09	0.81	0.58	5.09	3.65
9	1	1	0.44	0.77	2.73	4.15	0.64	0.77	3.99	4.84
11	− 1	0	0.85	0.80	5.32	5.01	0.68	0.67	4.28	4.19
14	0	0	0.82	0.72	5.13	4.53	0.53	0.53	3.33	3.28
16	1	− 1	0.00	0.00	0.00	0.00	1.39	1.32	8.72	8.28
17	1	0	0.94	1.14	7.84	7.09	0.45	0.58	2.81	3.65
18	1	1	0.86	0.77	3.35	4.15	0.57	0.77	3.6	4.84
20	− 1	0	0.79	0.80	4.92	5.01	0.67	0.67	4.17	4.19
23	0	0	0.43	0.72	2.68	4.53	0.54	0.53	3.35	3.28
25	1	− 1	0.00	0.00	0.00	0.00	1.46	1.32	9.11	8.28
26	1	0	1.26	1.14	7.54	7.09	0.49	0.58	3.05	3.65
27	1	1	1.02	0.77	6.38	4.15	1.11	0.77	6.92	4.84

### Surface response and optimal yeast growth

The surface response is shown in the form of three-dimensional graphics, which reveals the variation of the experimental response in the function of alterations in the levels of two selected variables: the temperature and pH for both YPD and SK media. The other variable is fixed at one determined level and the equation has been rearranged to generate its surface response (Shahabadi and Reyhani 2014).

## Results

### Screening for optimal physical parameters for protein production

The obtained biomasses of *Y. lipolytica* strains enriched in protein and amino acids exhibit different sensitivities to temperature and pH. This trial evaluated the effect of different values of pH (from 5.0 to 7.0) and temperature (from 20 to 30 °C) (Table 1). The optimal parameters for the cultures in two trial media (YPD medium and biofuel production waste) were screened and indicated that the temperature (X_1_) and pH (X_2_) had an apparent influence on *Y. lipolytica* protein-enriched production (Table 2). Factionary matrix planning (Tables 3, 4) was constructed from complete factorial planning based on two variables X_1_ and X_2_.

**Table 4:** Experimental design with experimental and predicted values of protein concentration in wet biomass of *Yarrowia lipolytica* strains obtained in cultivation on YPD medium

Code			YPD medium							
			TN (ATCC)		TP (ATCC)		TN (A101)		TP (A101)	
No.	X_1_	X_2_	Exp.	Pred.	Exp.	Pred.	Exp.	Pred.	Exp.	Pred.
2	− 1	0	1.4	1.45	8.73	8.97	1.51	1.53	9.47	9.60
5	0	0	1.42	1.37	8.85	8.55	1.78	1.67	11.1	10.40
7	1	-1	1.62	1.87	10.11	11.66	1.76	1.85	11.02	11.57
8	1	0	1.78	1.93	11.12	12.03	1.62	1.57	7.27	8.38
9	1	1	1.64	1.50	10.27	9.35	1.41	1.54	8.8	9.60
11	− 1	0	1.55	1.45	9.49	8.97	1.56	1.53	9.78	9.60
14	0	0	1.32	1.37	8.28	8.55	1.66	1.67	10.37	10.40
16	1	− 1	1.97	1.87	12.31	11.66	1.85	1.85	11.58	11.57
17	1	0	2.07	1.93	12.93	12.03	1.52	1.57	9.5	8.38
18	1	1	1.48	1.50	9.23	9.35	1.5	1.54	9.36	9.60
20	− 1	0	1.39	1.45	8.68	8.97	1.53	1.53	9.54	9.60
23	0	0	1.36	1.37	8.53	8.55	1.56	1.67	9.74	10.40
25	1	− 1	2.01	1.87	12.55	11.66	1.94	1.85	12.11	11.57
27	1	1	1.37	1.50	8.55	9.35	1.7	1.54	10.65	9.60

TN, total nitrogen; TP, total protein; Exp., experimental values; Pred., predicted values approximated from the Eq. (1); ATCC, *Y. lipolytica* ATCC 9793; A-101, *Y. lipolytica* A-101; YPD, YPD medium; SK, SK medium (biofuel production waste)

**Fig. 1:**
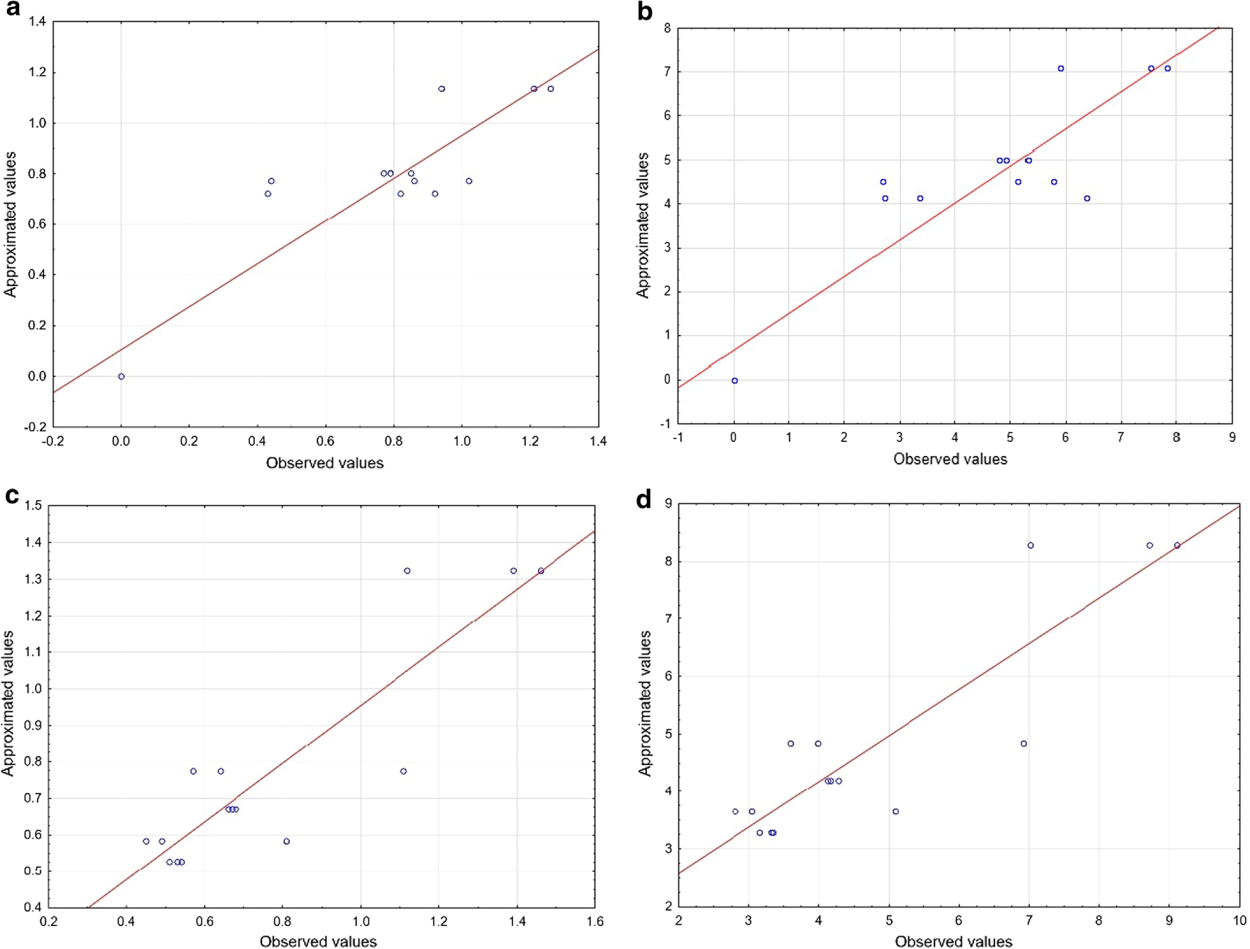
Correlation of observed values versus approximate values of TN and TP for *Yarrowia lipolytica* strains cultured on the YPD medium. **a** TN (*Y. lipolytica* ATCC 9793); **b** TP (*Y. lipolytica* ATCC 9793); **c** TN (*Y. lipolytica* A-101); **d** TP (*Y. lipolytica* A-101). *TN* total nitrogen, *TP* total protein

Diagnostic plots for the YPD medium were drawn to judge the model adequacy and clarify the signs of any problems in the experimental data. The plot of observed experimental response versus predicted response is shown in Fig. 1a–d. Herein, there are experimental values, and the approximate correlation difference was R^2^ = 0.8474 (R^2^adj. = 7864) for TN (*Y. lipolytica* ATCC 9793), R^2^ = 0.8399 (R^2^adj. = 7758) for TP (*Y. lipolytica* ATCC 9793), R^2^ = 0.7959 (R^2^adj. 0.7143) for TN (*Y. lipolytica* A-101), and R^2^ = 0.7982 (R^2^adj. = 0.7175) for TP (*Y. lipolytica* A-101).

These values of the R^2^ coefficients indicate good model matching.

On the basis of the response surface methodology and multivariate regression Eq. (1), dependencies of the dependent variables for the YPD medium were determined as shown below:

$$\begin{aligned}\text{TN} \;(\text{ATCC}) & = - 23.26 - 0.46 * (Temp) + 0.01 * {(Temp)^2}\\&\quad + 9.39 * pH - 0.75 * {\left( {pH} \right)^2}\end{aligned}$$

$$\begin{aligned}{\text{TP}} \;(\text{ATCC}) & = -155.48 - 2.84 * (Temp) + 0.06 \\&\quad* {(Temp)^2} + 62.24 * pH - 5.01 * {\left( {pH} \right)^2}\end{aligned}$$

$$\begin{aligned}{\text{TN}}\; (\text{A}101) & = 21.63 - 0.21 * (Temp) + 0.004 * {(Temp)^2}\\&\quad - 5.86 * pH + 0.47 * {\left( {pH} \right)^2}\end{aligned}$$

$$\begin{aligned}{\text{TP}} (\text{A}101) & = 135.78 - 1.34 \times (Temp) + 0.03 * {(Temp)^2} \\&\quad- 36.64 * pH + 2.91 * {\left( {pH} \right)^2}\end{aligned}$$

where ATCC is *Y. lipolytica* ATCC 9793 and A101 is *Y. lipolytica* A-101.

**Fig. 2:**
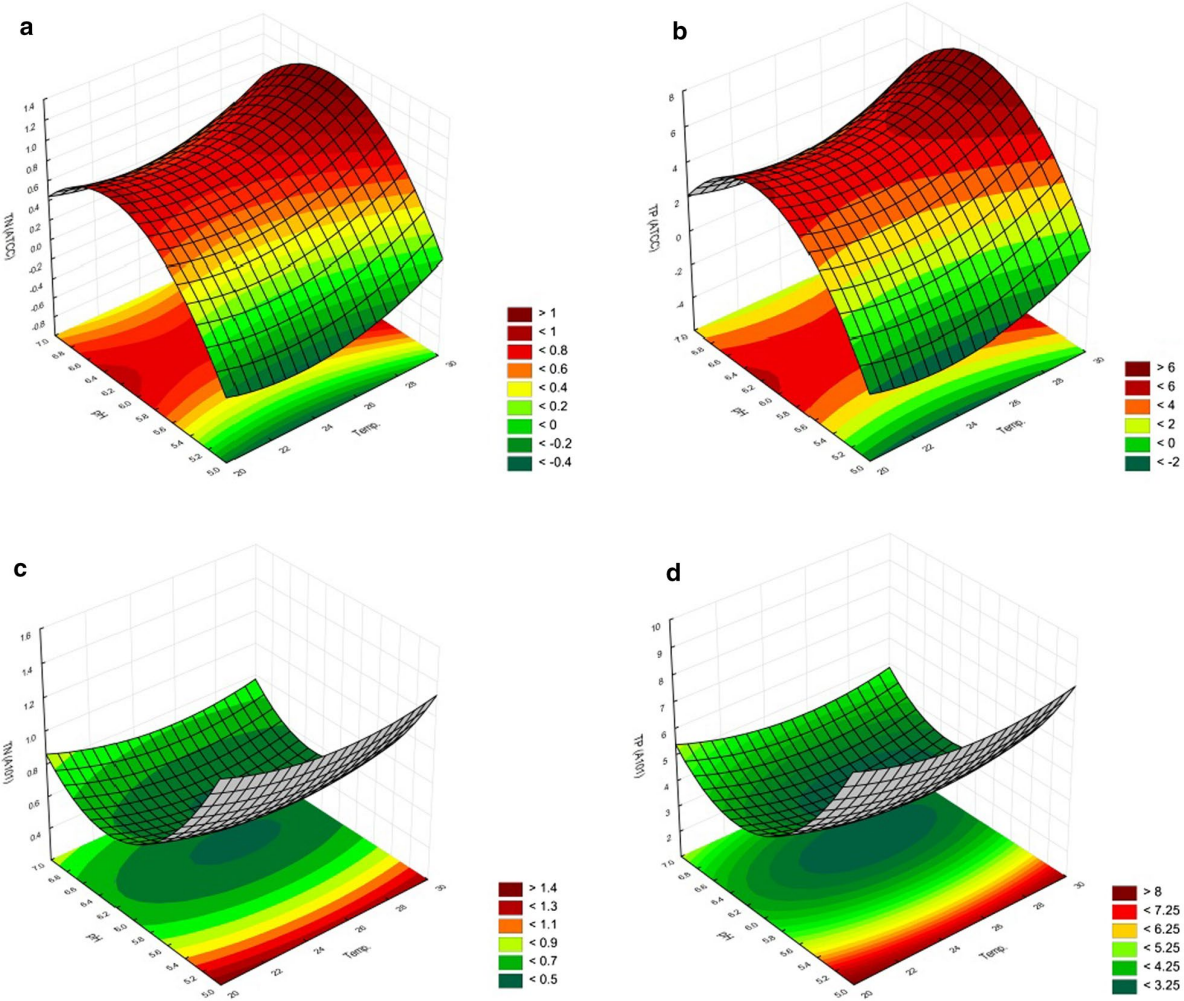
Response surface plots showing individual and interaction effects of the following variables: temperature and pH on TN and TP levels for *Y. lipolytica* ATCC 9793 and *Y. lipolytica* A-101 strains cultured on biofuel waste (the SK medium). **a** TN (*Y. lipolytica* ATCC 9793), **b** TP (*Y. lipolytica* ATCC 9793), **c** TN (*Y. lipolytica* A-101), **d** TP (*Y. lipolytica* A-101). TN, total nitrogen; TP, total protein

Three-dimensional (3D) response surface plots were drawn to illustrate the individual and interactive consequences of temperature and pH (Fig. 2). Each 3D plot shows the effects of two variables for both *Y. lipolytica* strains grown on biofuel waste (the SK medium). For the *Y. lipolytica* ATCC 9793 strain, the approximated highest TN content on the SK medium (biofuel waste) occurred at pH between 6.0 and 6.6 (Fig. 2a, b). The “saddle” graphs indicate the unimportant influence of temperature on the experiment result, though the pH value was statistically significant (although TN increased from 26 °C and over). In the experimental conditions, the highest TN value was obtained for pH 6.0 and the temperature of 30 °C. It was 1.14% ± 0.17 of wet biomass. In contrast, the use of *Y. lipolytica* A-101 and biofuel waste at the temperature of 30 °C and pH 5.0 significantly increased the amount of protein in the biomass (coded value, 0.5) (Fig. 2c, d). However, the TN content decreased with the increase in the pH value, the highest approximated values occurred at pH < 5.0, while the lowest values of the determined TN parameter occurred in the pH range from 6.2 to 6.5. The highest TN = 1.32 ± 0.18% was obtained for cultures at pH 5.0 and 30 °C.

In our work, we saw statistically significant (*P* < 0.05) high correlations between TN and TP content for *Y. lipolytica* ATCC 9793 (where R^2^ = 0.9449) and *Y. lipolytica* A-101 (where R^2^ = 0.9999), respectively. The highest TP values in wet biomass were obtained experimentally at the temperature of 30 °C and pH 6.0 for the ATCC 9793 strain (7.09% ± 1.05) and pH 5.0 for the A-101 strain (8.28% ± 1.11).

**Fig. 3:**
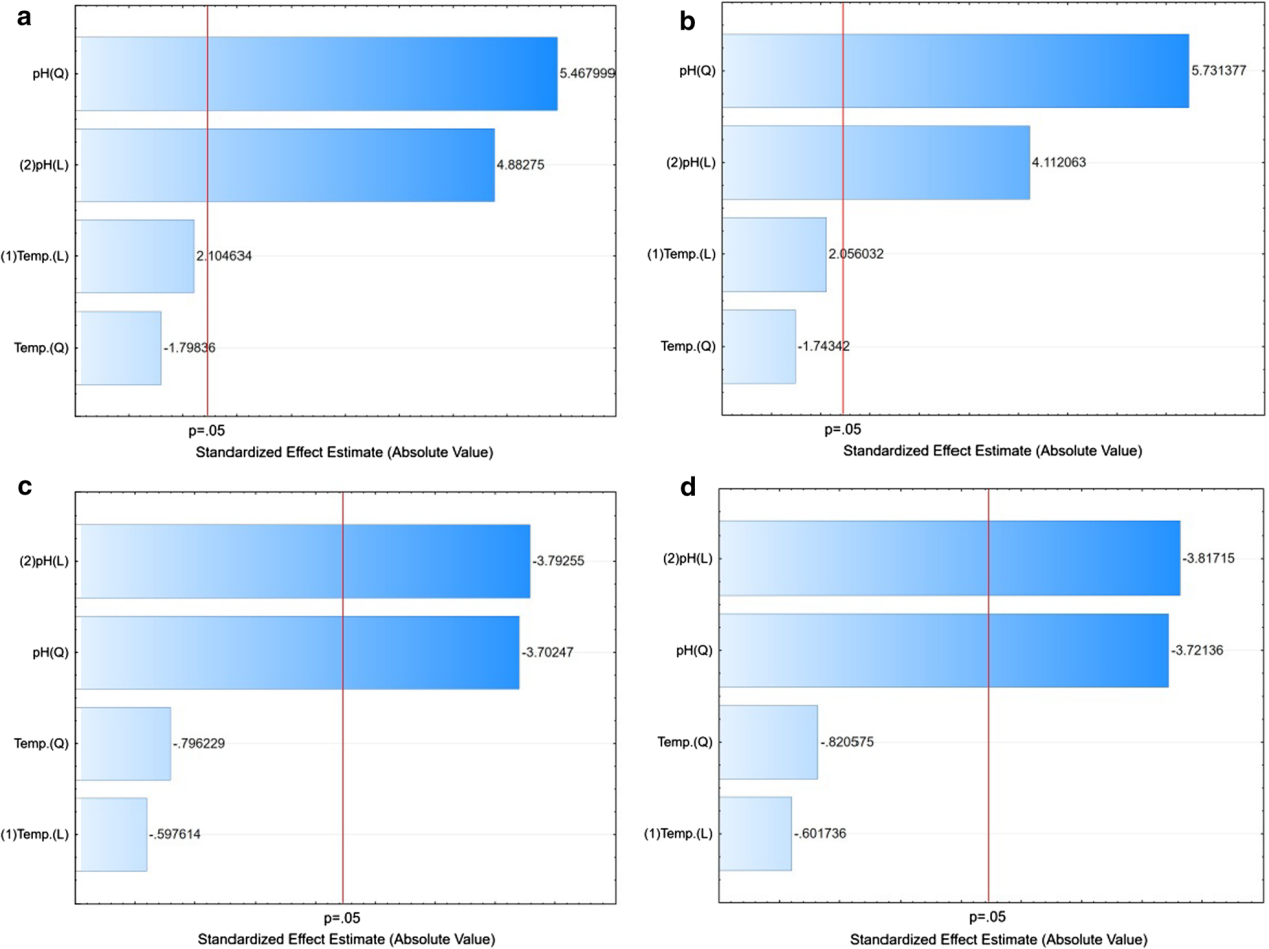
Pareto chart showing the effect of different culture parameters (variables) on protein-enriched biomass from the YPD medium based on the observation of the fractional factorial design. **a** TN (*Y. lipolytica* ATCC 9793); **b** TP (*Y. lipolytica* ATCC 9793); **c** TN (*Y. lipolytica* A-101); **d** TP (*Y. lipolytica* A-101); TN, total nitrogen; TP, total protein

The Pareto chart (Fig. 3a–d) showed that the model needed adjustment in terms of pH, and at the temperature of 30 °C, a variation towards lower pH was found to be necessary. In optimal conditions, higher values of TN and TP were obtained for the *Y. lipolytica* A-101 strain. In the ATCC 9793 strain, no cell growth was observed at pH 5.0.

For comparison, the culture was also cultivated on the YPD medium with the same dependent and independent variables. The mean values of TN and TP for the *Y. lipolytica* ATCC 9793 and A-101 strains obtained in cultivation on the YPD medium in relation to pH and temperature are presented in Table 2. For strain *Y. lipolytica* ATCC 9793, the highest values of TN and TP in wet biomass were obtained at the temperature of 30 °C and pH 6.0, with 1.93% ± 0.21 and 12.03% ± 1.28, respectively. The influence of independent variables on TN and TP values and approximate values are shown in Table 4.

**Fig. 4:**
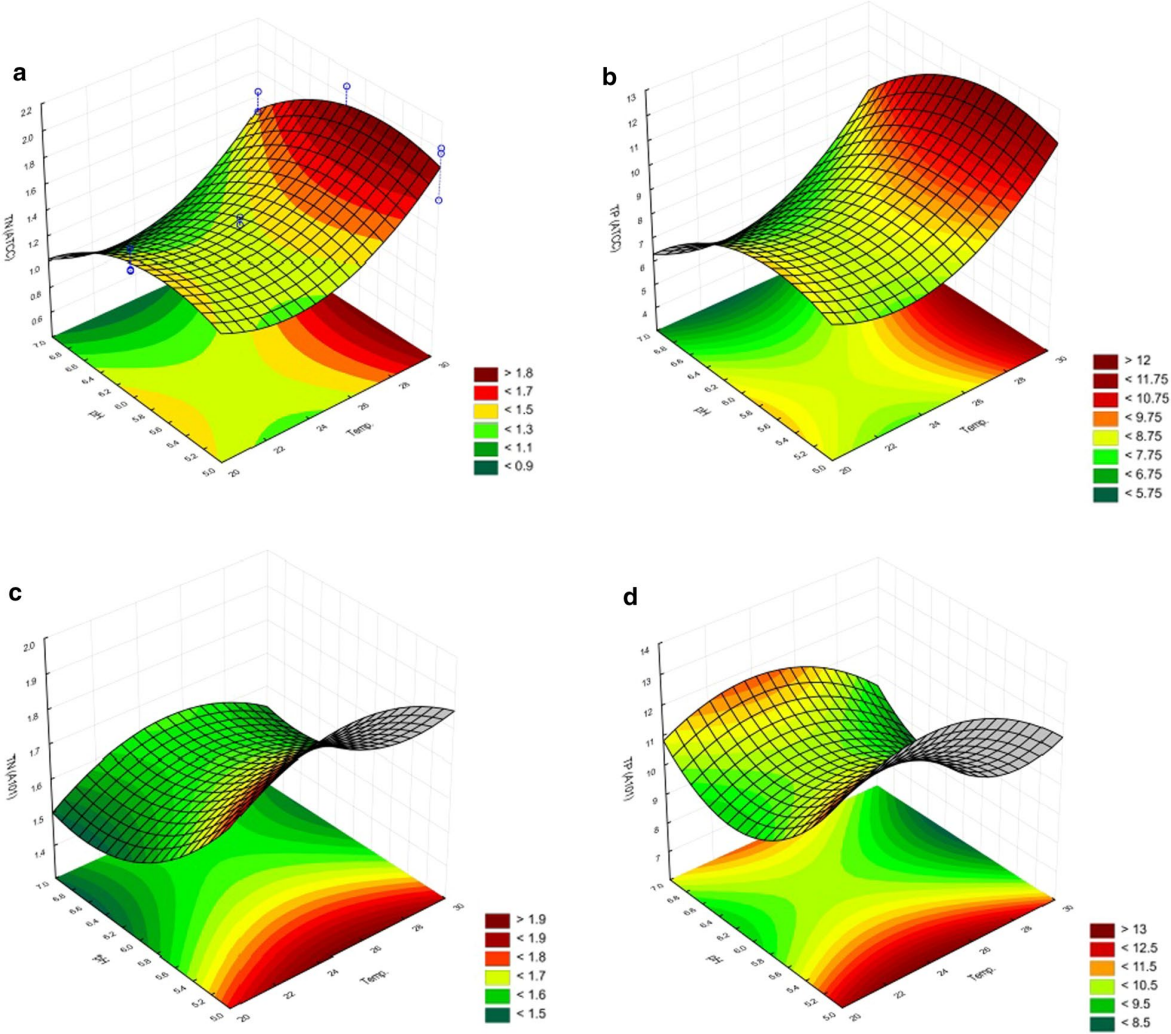
Surface response model of the influence of pH and temperature on TN and TP values for strains *Y. lipolytica* ATCC 9793 and *Y. lipolytica* A-101 cultivated on the YPD medium. **a** TN(*Y. lipolytica* ATCC 9793), **b** TP(*Y. lipolytica* ATCC 9793), **c** TN(*Y. lipolytica* A-101), **d** TP(*Y. lipolytica* A-101). TN, total nitrogen; TP, total protein

**Fig. 5:**
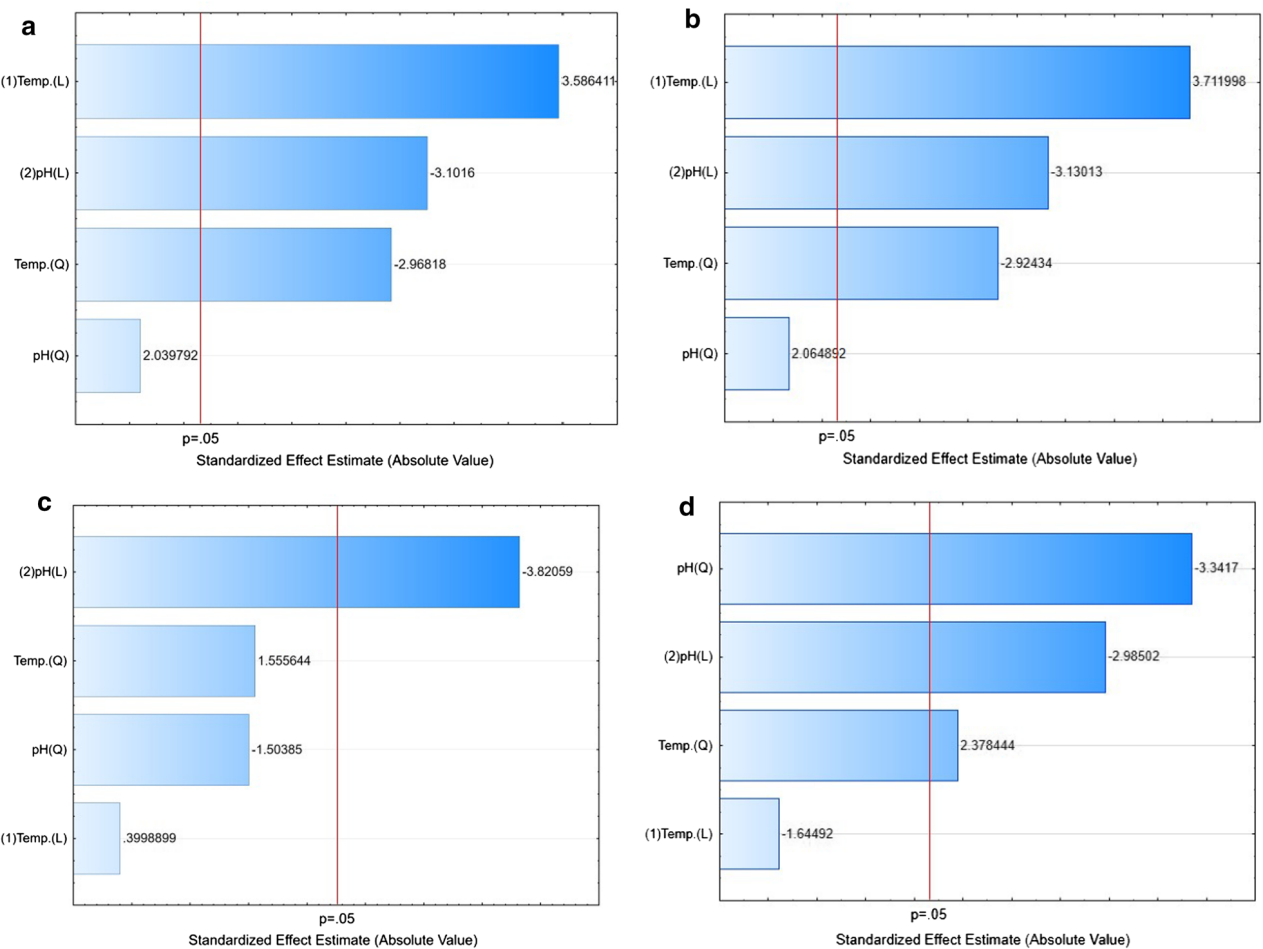
Pareto chart showing the effect of different culture parameters (variables) on protein-enriched biomass from biofuel waste (the SK medium) based on the observation of the fractional factorial design. **a**TN (*Y. lipolytica* ATCC 9793); **b** TP (*Y. lipolytica* ATCC 9793); **c** TN (*Y. lipolytica* A-101); **d** TP (*Y. lipolytica* A-101); TN, total nitrogen; TP total protein

As presented in Fig. 4, the levels of TN and TP for strain *Y. lipolytica* ATCC 9793 were closely related to the temperature of the cultivation. For *Y. lipolytica* ATCC 9793, the graphs show the effect of both parameters on the TN and TP values. However, for the *Y. lipolytica* A-101 strain, likewise for the SK medium (biofuel waste), such values were connected with the pH levels. The influence of independent values on the examined parameters is presented on Pareto charts presented in Fig. 5. The assessment of the influence of independent variables on the TN and TP values is represented by the response surface plot in Fig. 4 and in the Pareto charts in Fig. 5. As presented in the charts (Fig. 5a–d), in cases a-b for *Y. lipolytica* ATCC 9793, the values of TN and TP were closely related to the temperature and pH at which the experiment was conducted.

### Amino acid production

In our experiments, we also estimated the ability of *Y. lipolytica* to produce amino acids at the most suitable temperature for SPC production (30 °C) and in variable culture conditions such as the composition of the medium and its pH value. Additional file 1: Table S1 summarizes the results of these experiments. Both yeast strains used (*Y. lipolytica* ATCC 9793 and A-101) produced all essential and conditionally essential amino acids. Furthermore, the content of essential amino acids at pH 5.0 and 6.0 is almost the same and not statistically different.

## Discussion

*Yarrowia lipolytica* is a very good producer of SCP and amino acids with a sufficient amount of vitamin B12 in its biomass when it grows on inexpensive biofuel production waste where glycerol and vegetable oil were used as carbon sources (Jach et al. 2017, 2018). Biofuel waste is a liquid oil substrate that is dispersed very easily with moderate agitation while solid fat materials (e.g. tallow) require considerable agitation (e.g. 1200 rpm) for dispersal in the growth medium (Papanikolaou et al. 2007). Additionally, *Y. lipolytica* growing on fatty substrates tends to metabolize poly-unsaturated fatty acids and is able to accumulate mono-unsaturated (e.g. oleic and linoleic acid) and saturated ones, producing cocoa-butter equivalents (Aggelis et al. 1997; Lopes et al. 2019; Papanikolaou et al. 2001, 2003; Zhao et al. 2016).

*Y. lipolytica* possesses hydrophobic cell surfaces. Therefore, it can stick to lipid molecules present in the hydrophobic medium and secretes emulsifiers and surfactants. Lipids often occur as triacylglycerols (TAGs) in the growth environment (Papanikolaou et al. 2002, 2007; Saygün et al. 2014). *Y. lipolytica* is capable of producing and releasing lipases that break down TAGs into fatty acids and glycerol (Thevenieau et al. 2010). However, the concentration of lipases declines in the stationary growth phase (Papanikolaou et al. 2007). Interestingly, it is enough to add an oil substrate such as waste cooking oil to the growth medium to induce a significant increase in extracellular lipase production by the yeast in comparison with oil-free cultures. Therefore, the addition of oil substrates to the yeast culture plays a role of a lipase inductor (Dominguez et al. 2010; Lopes et al. 2019). Thus, the yeast is able to utilize lipid substrates.

The ability to accumulate storage lipids allows sustaining the growth of the yeast even in the absence of an extracellular carbon source (Daskalaki et al. 2019). Yarrowia cells can use storage lipids for varied metabolic activities, including the production of maintenance energy (Dourou et al. 2018). Noteworthy, the biosynthesis of protein or cellular polysaccharides in *Y. lipolytica* cells is competitive to the lipid accumulation (Daskalaki et al. 2019; Dourou et al. 2018). Daskalaki et al. (2019) observed significant lipid degradation, which coincided with production of fat-free biomass and an increase in protein content. Moreover, the lipogenic ability also depends on nitrogen availability (Bellou et al. 2016). Nitrogen starvation in the culture medium leads to a rapid reduction of intracellular AMP, which causes inhibition of mitochondrial AMP-activated isocitrate dehydrogenase (ICDH). This inhibition is decisive in signaling the first step of lipogenesis, because the disturbance of the Krebs cycle induces accumulation of intra-mitochondrial citric acid, causing excretion of citric acid to the cytoplasm instead of malate. Next, citric acid is hydrolyzed to acetyl-CoA and oxaloacetate by ACL in the cytoplasm, causing induction of carbon transition towards the metabolic pathway competitive to lipogenesis, e.g. production of protein and amino acids. Interestingly, transcription of genes for ACL and ICDH is observed in both non-oleaginous and oleaginous conditions (Bellou et al. 2016; Dourou et al. 2018).

The ability to produce SCP and amino acids is not a static property, and it can be considerably affected by fermenting-process parameters. This study was focused mainly on improving protein production by the *Y. lipolytica* A-101 strain at different temperatures (from 20 °C to 30 °C) and pH values (from 5.0 to 7.0). Some studies have proven that *Y. lipolytica* is able to produce different quantities of lipids, depending on the hydrophobic media and fermenting conditions (Papanikolaou et al. 2002, 2003; Zhao et al. 2016). Papanikolaou et al. (2002) showed that accumulation of lipids during primary anabolic growth was largely influenced by the medium pH and the incubation temperature. Additionally, Zhao et al. (2016) observed that as the temperature increased (from 20 °C to 35 °C), the biomass yield halved while the lipid content increased. However, in contrast to these actions, Daskalaki et al. (2019) used adaptive laboratory evolution (ALE) strategies to derive new highly productive strains. After 77 generations, they obtained a population that was capable of lipid accumulation at a 30% higher level than the starting *Y. lipolytica* strain.

It was also indicated that optimization experiments using statistical experimental-design techniques are the most appropriate for selecting significant variables for obtaining improved levels of yield (Polpass et al. 2013; Shahabadi and Reyhani 2014; Lopes et al. 2019). Using statistical optimization methods, we have shown that a small degree of manipulation of the fermentation parameters can exert significant effects on the production of SCP by *Y. lipolytica* without making changes in the culture medium composition. In contrast, Hezarjaribi et al. (2016) used statistical techniques to manipulate and optimize medium constituents to increase protein production. These researchers applied the full-factorial-methodology approach for optimization of the medium for SCP production by *Saccharomyces cerevisiae,* achieving the level of protein comparable to our results. In turn, Lopes et al. (2019) used the statistical experimental design based on the Taguchi method estimating simultaneously the effect of initial medium pH and manipulation of the medium and constituent concentration on lipase production by a *Y. lipolytica* strain. In this case, pH proved to be the most significant parameter; however, the interaction between the medium and constituent concentration had the highest impact on production of lipase.

In this study, we demonstrated significant mutual interactions between the temperature, pH, medium, and strain. When the strains were grown on biofuel waste (the SK medium) where glycerol and vegetable oils were used as a carbon source, the temperature was statistically insignificant in terms of the nutrients obtained. However, the higher TN content in *Y. lipolytica* ATCC 9793 was directly proportional to the increasing temperature. In contrast, for *Y. lipolytica* A-101 growing on the SK medium (biofuel waste), the only significant parameter was the pH value. In this experiment, the temperature did not affect the determined parameter. These results coincided with the experimental results in which the highest TN and TP were obtained for cultures at pH 5.0 and 30 °C. The optimal conditions for the TN and TP content were identical.

This study emphasized the statistical experimental analysis using the graphic method (Pareto chart) and the interpretation of the interactions among variables. The results can be attributed to the levels of variables that were the closest to the optimal level. The distance between the lowest and highest quantitative levels was sufficient to promote significant differences. Therefore, for both tested strains grown on biofuel waste, the increase in the TN and TP content was affected statistically significantly by the environment (Fig. 3). Here, the pH value was the statistically significant parameter (*P* < 0.05). However, for *Y. lipolytica* A-101 (Fig. 3c, d), this relationship had the opposite sign “-”. This means that the value of the dependent variables TN and TP increased at a lower pH.

While the cultures were cultivated on the YPD medium where peptone served as a carbon source, the highest values of TN and TP were obtained with the same independent variables as in cultivation on the SK medium. However, the levels of TN and TP for strain *Y. lipolytica* ATCC 9793 were closely related to the temperature and pH of the cultivation. With an increase in the temperature, the dependent variable values rose. Moreover, as shown by Katre et al. (2012), using an expensive substrate (e.g. glucose or peptone) as a carbon source limited its use for production of planned products. Therefore, the ability of *Y. lipolytica* strains to produce biomass rich in various nutritional components on available cheap carbon sources like oily wastes is highly desired. Furthermore, biodegradation of these wastes is very important for environmental protection (Katre et al. 2012; Saygün et al. 2014; Vasiliadou et al. 2018; Tzirita et al. 2018). Additionally, there are no generated wastes according to the take-make-dispose concept (Lopes et al. 2019).

For the *Y. lipolytica* A-101 strain, likewise for the SK medium (biofuel waste), the TN values were mainly dependent on pH levels. With regard to the TP values, the temperature of the cultivation had an additional influence besides the pH levels. Through statistical analysis, we confirmed the regularity of our previous research plan. It consisted of five different measuring systems wherein we obtained information about the influence of culture parameters such as pH and temperature on yeast growth (Jach et al. 2017). This effect allowed the development of a multivariate regression equation. The statistical analysis showed that protein-enriched *Y. lipolytica* A-101 biomass from biofuel waste is best obtained at the temperature of 30 °C and pH 5.0. In these conditions, the strain produced 40-50% of protein in its dry cell weight (Jach et al. 2017). Moreover, the amino acid level obtained was sufficient and not statistically significant from the quantity of amino acids obtained at the same temperature and pH 6.0. Additionally, we earlier found that vitamin B12-enriched biomass was produced from biofuel waste by *Y. lipolytica* A-101 grown at the temperature of 30 °C and pH 5.0 in a biofermentor (Jach et al. 2018). It is noteworthy that temperature and pH also affect lipase activity. Dominguez et al. (2010) showed maximum activities of enzymes at temperature values between 30 and 40 °C and the highest stability in acidic environments (pH 5.0), while a dramatic fall in stability was observed at pH values over 6.5. Therefore, protein production occurs under the same conditions in which lipases work most efficiently.

The fractional factorial design turned out to be a method that can be used not only in preparation of an experiment plan but also in interpretation and drawing conclusions from previously acquired lab results. Although it was not possible to invert the X’X matrix, we managed to assess linear (mainly) and square effects of culture parameters, e.g. temperature and pH. Detailed optimization with additional input parameters (independent or dependent variables), e.g. biomass production or other measurable breeding parameters, will be the subject of our further research.

Based on the predicted and experimental data, we found that using biofuel waste for obtaining protein-enriched biomass of *Yarrowia lipolytica* requires a precise control of only one parameter, i.e. the pH level. This appears to be a good solution for industrial-scale production. Thus, Yarrowia dried biomass derived from the yeast culture grown on biofuel waste can be regarded as a cheap, safe, and good source of essential amino acids and protein with an appropriate amount of vitamin B12, particularly for people who avoid eating meat (e.g. vegan and vegetarian) or live in poor regions and places with deficiency of food sources. Nevertheless, for protein-enriched Yarrowia biomass to be commercially successful in the future, food produced from the yeast biomass must be similar in taste to the food already known and available in the local market.

## Supplementary information

**Additional file 1: Table S1.** Mean content of amino acids in wet biomass of *Yarrowia lipolytica* strains growing on various culture conditions on laboratory scale (grams of amino acid per 100 grams of protein or 16 grams of nitrogen at up to 5% of standard deviation).

## Data Availability

The datasets supporting the conclusions of this article are available in the FigShare repository https://figshare.com/s/3c9782529b6c6e28d4b3.
