# Supplementary material for: Statistical evaluation of growth parameters in biofuel waste as a culture medium for improved production of single cell protein and amino acids by Yarrowia lipolytica
Source: AMB Express. 2020 Feb 18;10:35. doi: 10.1186/s13568-020-00968-x (PMC7028882; doi:10.1186/s13568-020-00968-x)
Supplement: Supplementary file 1 — Additional file 1: Table S1. Mean content of amino acids in wet biomass of Yarrowia lipolytica strains growing on various culture conditions on laboratory scale (grams of amino acid per 100 grams of protein or 16 grams of nitrogen at up to 5% of standard deviation). [file 13568_2020_968_MOESM1_ESM.docx]

AMB Express

Statistical evaluation of growth parameters in biofuel waste as a culture medium for improved production of single cell protein and amino acids by *Yarrowia lipolytica*

Monika Elżbieta Jach^1*^, Tomasz Baj^2^, Marek Juda^3^, Robert Świder^1^, Barbara Mickowska^4^, Anna Malm^3^

^1^The John Paul II Catholic University of Lublin, Department of Molecular Biology, 1i Konstantynów Street, 20-708 Lublin, Poland, monijach@kul.lublin.pl, swider.rob@gmail.com

^2^Medical University of Lublin, Chair and Department of Pharmacognosy, 1 Chodzki Street, 20-093 Lublin, Poland, *tbaj@pharmacognosy.org*

^3^Medical University of Lublin, Department of Pharmaceutical Microbiology, 1 Chodzki Street, 20-093 Lublin, Poland, marek.juda@onet.pl, anna.malm@umlub.pl

^4^*T*he Agricultural University of Cracow, Faculty of Food Technology, Malopolska Centre of Food Monitoring, Balicka Street 122, 30-149 Cracow, Poland, bmickowska@ar.krakow.pl

*****Corresponding author. Tel.: +48 81 475 5432, fax: +48 81 4454611, e-mail address: monijach@kul.lublin.pl (M.E.Jach),

ORCID ID 0000-0002-4932-3260

Table S1. Mean content of amino acids in wet biomass of *Yarrowia lipolytica* strains growing on various culture conditions on laboratory scale (grams of amino acid per 100 grams of protein or 16 grams of nitrogen at up to 5% of standard deviation).

| Amino acid | YPG, 30°C, pH 4.0 | | SK, 30°C, pH 4.0 | | YPG, 30°C, pH 5.0 | | SK, 30°C, pH 5.0 | | YPG, 30°C, pH 6.0 | | SK, 30°C, pH 6.0 | | YPG, 30°C, pH 7.0 | | SK, 30°C, pH 7.0 | |
| --- | --- | --- | --- | --- | --- | --- | --- | --- | --- | --- | --- | --- | --- | --- | --- | --- |
|  | **ATCC** | **A-101** | **ATCC** | **A-101** | **ATCC** | **A-101** | **ATCC** | **A-101** | **ATCC** | **A-101** | **ATCC** | **A-101** | **ATCC** | **A-101** | **ATCC** | **A-101** |
| Aspartic acid# | 8.0 | 8.4 | x | 5.6 | 8.3 | 8.7 | x | **9.8** | 6.8 | 9.5 | 8.4 | 10.1 | 9.8 | 7.5 | 10.2 | 7.6 |
| Threonine* | 4.2 | 4.5 | x | 2.9 | 3.7 | 4.6 | x | **5.1** | 3.4 | 4.6 | 4.3 | 5.1 | 5.3 | 4.0 | 5.3 | 4.0 |
| Serine | 3.4 | 3.7 | x | 2.6 | 3.2 | 3.9 | x | **4.6** | 3.1 | 4.2 | 4.3 | 4.8 | 4.9 | 3.8 | 4.6 | 3.5 |
| Glutamic acid# | 8.9 | 9.4 | x | 6.7 | 9.5 | 10.5 | x | **10.8** | 8.9 | 13.0 | 11.4 | 12.9 | 12.0 | 8.1 | 12.2 | 9.8 |
| Proline | 3.2 | 3.6 | x | 2.6 | 3.3 | 3.8 | x | **4.5** | 2.9 | 3.8 | 3.9 | 4.5 | 5.1 | 3.6 | 4.2 | 3.6 |
| Glycine | 4.3 | 4.5 | x | 3.0 | 4.1 | 4.7 | x | **5.3** | 3.5 | 5.0 | 4.1 | 5.2 | 5.2 | 3.9 | 5.3 | 4.2 |
| Alanine | 5.4 | 6.0 | x | 4.2 | 5.0 | 6.1 | x | **7.8** | 5.2 | 8.1 | 8.6 | 8.9 | 8.6 | 6.6 | 6.9 | 5.9 |
| Valine* | 4.6 | 5.1 | x | 3.4 | 4.3 | 5.3 | x | **5.9** | 3.8 | 5.5 | 4.7 | 5.9 | 6.2 | 4.8 | 5.7 | 4.6 |
| Isoleucine* | 3.6 | 4.0 | x | 2.7 | 3.3 | 4.1 | x | **4.8** | 3.1 | 4.4 | 3.8 | 4.8 | 4.9 | 3.9 | 4.5 | 3.6 |
| Leucine* | 5.2 | 5.9 | x | 4.0 | 48 | 6.1 | x | **7.0** | 4.9 | 7.2 | 6.3 | 7.9 | 8.1 | 6.2 | 6.7 | 5.5 |
| Tyrosine | 4.9 | 6.3 | x | 5.5 | 3.0 | 5.3 | x | **11.8** | 3.1 | 7.2 | 9.1 | 13.9 | 9.2 | 9.9 | 4.4 | 4.2 |
| Phenylalanine* | 3.6 | 3.7 | x | 2.5 | 32 | 3.9 | x | **4.4** | 2.6 | 4.4 | 3.5 | 4.4 | 4.6 | 3.4 | 4.4 | 3.5 |
| Histidine | 2.3 | 2.6 | x | 1.7 | 2.4 | 2.9 | x | **3.0** | 2.2 | 3.6 | 2.3 | 3.0 | 3.4 | 3.0 | 3.1 | 2.9 |
| Lysine* | 6.5 | 7.1 | x | 4.6 | 6.0 | 7.3 | x | **7.8** | 5.8 | 7.8 | 6.3 | 8.0 | 76 | 5.9 | 8.3 | 6.2 |
| Arginine | 4.0 | 4.6 | x | 3.1 | 3.6 | 4.7 | x | **5.5** | 3.6 | 5.0 | 4.4 | 5.5 | 5.5 | 4.3 | 5.2 | 4.1 |
| Cysteine | 0.7 | 1.0 | x | 0.6 | 0.7 | 1.0 | x | **1.0** | 0.7 | 1.1 | 11 | 1.2 | 1.2 | 1.0 | 1.1 | 1.1 |
| Methionine* | 1.1 | 1.4 | x | 0.9 | 1.0 | 1.4 | x | **1.6** | 1.0 | 1.5 | 1.3 | 1.6 | 19 | 1.7 | 1.7 | 1.4 |
| Tryptophan* | 1.2 | 0.7 | x | 1.0 | 1.1 | 0.4 | x | **1.9** | 0.7 | 0.8 | 0.7 | 1.2 | 2.0 | 0.7 | 0.8 | 0.3 |
| **Total** | 75.1 | 82.5 | - | 57.6 | 70.5 | 84.7 | - | **102.6** | 65.3 | 96.7 | 88.5 | 108.9 | 105.5 | 82.3 | 94.6 | 76.0 |

x – indicates no growth; ATCC – *Y. lipolytica* ATCC 9773; A-101 – *Y. lipolytica* A-101; YPD – YPD medium; SK – SK medium (biofuel production waste); ^#^During analysis asparagine and glutamine turned to aspartic acid and glutamic acid respectively and in these forms are determined, so results Asp=Asp+Asn, and Glu=Glu+Gln.Each result is the average of three independent measurements.
